# Protective Effects of *Misgurnus mizolepis* Protein Hydrolysate against Atropine-Induced Dry Eye Disease

**DOI:** 10.4014/jmb.2509.09010

**Published:** 2025-11-26

**Authors:** Da Hye Kim, EunJin Bang, Seon Yeong Ji, Hyun Hwangbo, Min Yeong Kim, Jung-Hyun Shim, Gi-Young Kim, You-Jin Jeon, Suengmok Cho, Yung Hyun Choi

**Affiliations:** 1 Department of Biochemistry, Dong-eui University College of Korean Medicine, Busan 47227, Republic of Korea; 2 Basic Research Laboratory for the Regulation of Microplastic-Mediated Diseases and Anti-Aging Research Center, Dong-eui University, Busan 47227, Republic of Korea; 3 Department of Biomedicine, Health & Life Convergence Sciences, BK21 Four, College of Pharmacy, Mokpo National University, Muan 58554, Republic of Korea; 4 Department of Marine Life Science, Jeju National University, Jeju 63243, Republic of Korea; 5 Department of Food Science and Technology and Institute of Food Science, Pukyong National University, Busan 48513, Republic of Korea

**Keywords:** Dry eye disease, *Misgurnus mizolepis*, protein hydrolysate, anti-inflammation, anti-oxidation, apoptosis

## Abstract

Dry eye disease (DED) is a multifactorial ocular disorder characterized by tear film instability, inflammation, and ocular surface damage. Although various therapeutic approaches are available, there remains a strong need for safer and more effective agents with clearly defined mechanisms of action. This study examined the protective effects of *Misgurnus mizolepis* protein hydrolysate (MMH) in both *in vitro* and *in vivo* models of DED. *In vitro*, pretreatment of air-dried human corneal epithelial cells with MMH attenuated oxidative stress and apoptosis. *In vivo*, oral administration of MMH to rats with atropine-induced DED restored tear secretion, preserved ocular tissue architecture, reduced immune cell infiltration, and downregulated inflammatory mediators in the cornea. Furthermore, MMH maintained tight junction proteins, suppressed pro-apoptotic signaling in the lacrimal gland, improved meibomian gland and goblet cell integrity, and mitigated neovascularization. Collectively, MMH demonstrated anti-inflammatory, anti-apoptotic, and tissue-protective effects, supporting its potential as a novel therapeutic candidate for DED.

## Introduction

*Misgurnus mizolepis* protein hydrolysate (MMH) is obtained *via* enzymatic hydrolysis of *M. mizolepis*, a freshwater fish species traditionally consumed in South Korea [1, 2]. Typically, protein hydrolysates from diverse natural sources are rich in low-molecular-weight peptides and amino acids, which are readily absorbed and exhibit high bioavailability [3, 4]. Numerous studies have demonstrated that fish protein hydrolysates possess bioactive properties, including antioxidant, anti-inflammatory, and antihypertensive activities [5-7]. For instance, loach protein hydrolysates prepared *via* papain digestion exhibit oxygen radical scavenging activity, and their antioxidant activity is correlated with antiproliferative activity in cancer cells [8]. Similarly, *Katsuwonus pelamis* hydrolysates exert nephroprotective effects by modulating interleukin (IL)-17 and tumor necrosis factor (TNF) signaling pathways [9]. Despite these findings, the physiological activities of hydrolysates derived from *M. mizolepis* remain unexplored.

Dry eye disease (DED) is a prevalent multifactorial ocular disorder characterized by tear film instability, inflammation, and ocular surface damage. Risk factors include aging, prolonged use of digital devices, air pollution, contact lens wear, and long-term medication use [10, 11]. Tears play an essential role in maintaining ocular surface integrity; disruptions in tear secretion or composition can cause inflammation, keratitis, conjunctivitis, and visual impairment [12-14]. The tear film is composed of mucin, aqueous, and lipid layers, secreted by conjunctival goblet cells, lacrimal glands, and meibomian glands, respectively [15, 16]. This finely balanced structure is vulnerable to both intrinsic and extrinsic insults, which may ultimately result in the development of DED.

Current therapeutic strategies for DED include artificial tears, anti-inflammatory agents, and omega-3 fatty acid supplementation [11, 17]. However, these treatments often show limited efficacy and may cause adverse effects with long-term use. Therefore, safer and more effective alternatives are urgently required. Oral administration of natural bioactive compounds has attracted attention due to their antioxidant, anti-inflammatory, and tissue-protective properties [18, 19]. The present study aimed to evaluate the protective and anti-inflammatory effects of orally administered MMH in both *in vitro* and *in vivo* DED models. This is the first report to investigate the ocular protective potential of *M. mizolepis* protein hydrolysate and offers novel evidence for its therapeutic applicability in DED management.

## Materials and Methods

### Cell Culture and Dry Eye Induction

Human corneal epithelial cells (hCECs; PCS-700-010, American Type Culture Collection, USA) were cultured in a 1:1 mixture of Keratinocyte Serum-Free Medium (Thermo Fisher Scientific, USA) supplemented with 25 mg/ml bovine pituitary extract and 2.5 μg/ml recombinant human epidermal growth factor, together with DMEM/F-12 medium (WelGENE, Republic of Korea) containing 10% fetal bovine serum and 1% penicillin-streptomycin (WelGENE). Cells were maintained in a humidified incubator at 37°C with 5% CO_2_. To simulate dry eye conditions *in vitro*, the culture medium was completely removed, and the cells were exposed to ambient air at room temperature (RT) for up to 5 min on a clean bench, as previously described [20].

### Preparation and Administration of MMH

MMH was kindly provided by the laboratory of Professor Yujin Jeon at Jeju National University (Republic of Korea). MMH preparation was performed according to the protocol described in a previous study, with minor modifications [21]. The sample was thoroughly washed and mixed with distilled water at a ratio of 1.5:1 (w/w; water:sample). Subsequently, enzymatic hydrolysis was performed at 50–60°C for 4 h using Alcalase 2.4 L FG (Novozymes, Denmark). The reaction was terminated by heating the mixture at 85°C for 10 min to inactivate the enzyme. The resulting hydrolysate was filtered, concentrated under reduced pressure, and lyophilized to obtain MMH in powder form.

### Cell Viability Assay

Cell viability was assessed using the Cell Counting Kit-8 (CCK-8; Abcam, UK), as per the manufacturer’s instructions [22]. Following treatment with MMH, cells were incubated with the CCK-8 solution for 30 min at 37°C. Subsequently, their absorbance was recorded at 460 nm using a Synergy HTX microplate reader (BioTek Instruments).

### Flow Cytometric Analysis of Reactive Oxygen Species (ROS) and Apoptosis

Intracellular ROS levels were quantified 30 min post-exposure to air-drying by staining the cells with 10 μM 5,6-carboxy-2',7'-dichlorodihydrofluorescein diacetate (DCF-DA; Thermo Fisher Scientific) for 30 min at 37°C [23]. For apoptosis detection, cells were stained 24 h after air-drying using an Annexin V-fluorescein isothiocyanate (FITC)/propidium iodide (PI) apoptosis detection kit (BD Biosciences, USA) [24]. Analyses were performed with an Accuri C6 flow cytometer (BD Biosciences).

### Quantitative Real-Time PCR (qRT-PCR)

Total RNA was extracted from the cells using TRIzol reagent (Thermo Fisher Scientific), and complementary DNA was synthesized with the SuPrimeScript RT Premix (GenetBio, Republic of Korea). Thereafter, qRT-PCR was performed using TOPreal SYBR Green qPCR PreMIX (Enzynomics, Republic of Korea) on a CFX Duet Real-Time PCR System (Bio-Rad, USA) [25]. Glyceraldehyde-3-phosphate dehydrogenase was used as the internal reference gene, and relative expression levels were determined using the 2^–ΔΔCt^ method. Primer sequences used in this experiment are provided in Table 1.

### *In Vivo* Model of DED

Six-week-old male Sprague–Dawley rats (220 ± 20 g; Koatech, Republic of Korea) were used to establish the DED model. The animals were housed in a controlled environment (23 ± 2°C; 55 ± 9% relative humidity) under a 12-h light/dark cycle, with *ad libitum* access to standard chow and water. All experimental procedures were approved by the Institutional Animal Care and Use Committee of Dong-eui University (approval number: R2024-003) and performed in compliance with the institutional guidelines. Following a 7-day acclimatization period, the rats were randomly assigned to six groups (*n* = 6 per group): control, DED, MMH low-dose (50 mg/kg/day; ML), MMH middle-dose (100 mg/kg/day; MM), MMH high-dose (200 mg/kg/day; MH), and positive control (lutein, 4 mg/kg/day; PC). DED was induced in each experimental group by applying 1% atropine sulfate (AP; Thermo Fisher Scientific) topically to both eyes three times daily for 21 days, in accordance with a previously published protocol [26]. Subsequently, MMH and lutein were administered orally once daily for 14 consecutive days. At the end of the treatment period, ocular surface parameters were assessed, and tissues (eyes and lacrimal glands) were harvested for further analysis.

### Corneal Fluorescein Staining

A 1% fluorescein sodium solution (Merck KGaA, Germany) was applied to the corneal surface. After gently rinsing away excess dye with saline, the cornea was examined under cobalt blue light to visualize fluorescence. Images were acquired, and the fluorescence intensity was quantified using ImageJ software (National Institutes of Health, USA).

### Measurement of Tear Secretion

Tear production in rats was evaluated using BIO Color Tear Test strips (Bio Optics, Republic of Korea), which were trimmed to appropriate sizes. Tear wetting length was recorded and expressed relative to the normal control group [27].

### Histological Analysis of Ocular Tissues

Ocular tissues, including the cornea, retina, conjunctiva, and lacrimal gland, were fixed in 4% paraformaldehyde (Merck KGaA), dehydrated through a graded ethanol series, cleared in xylene, and embedded in paraffin (Sakura Finetek USA, Inc., USA). Subsequently, the paraffin-embedded tissues were sectioned at 5 μm thickness using a microtome (CM1860; Leica Biosystems, Germany) and mounted on gelatin-coated slides [27]. In parallel, a subset of tissues was also fixed in 4% paraformaldehyde and cryoprotected by sequential immersion in 10% and 20%sucrose solutions for 6 h each, followed by 30% sucrose overnight. Thereafter, cryoprotected samples were embedded in optimal cutting temperature compound (Sakura Finetek) and frozen at −20°C to prepare frozen blocks. Cryosections (10 μm thick) were obtained using a cryostat (CM1860; Leica Biosystems and mounted on gelatin-coated slides [28]. All paraffin-embedded tissue sections were rehydrated using xylene and a graded series of ethyl alcohol solutions prior to staining.

### Hematoxylin and Eosin (H&E) Staining

Paraffin sections were deparaffinized, rehydrated, and stained with hematoxylin and eosin (YD Diagnostics Co., Republic of Korea) to evaluate general tissue morphology [29].

### Immunofluorescence Staining

Cryosections were permeabilized with phosphate-buffered saline (PBS) containing 0.1% Triton X-100 (PBS-T) and blocked with 5% bovine serum albumin (Thermo Fisher Scientific) in PBS-T for 1 h at RT. The sections were then incubated overnight at 4°C with primary antibodies against Claudin-1, apoptosis-associated speck-like protein containing a Apoptosis-associated speck-like protein containing a caspase recruitment domain, Annexin V (Santa Cruz Biotechnology, USA), zonula occludens-1 (ZO-1; Thermo Fisher Scientific), Nucleotide-binding oligomerization domain-like receptor (NLR) family pyrin domain containing 3 (NLRP3), and nuclear factor kappa-light-chain-enhancer of activated B cells (NF-κB; Cell Signaling Technology, USA). After washing with PBS-T, fluorophore-conjugated secondary antibodies (BioActs, Republic of Korea) were applied for 1 h at RT. Subsequently, the nuclei were counterstained with 2.5 μg/ml 4',6-diamidino-2-phenylindole dihydrochloride (DAPI; Thermo Fisher Scientific), and slides were mounted using Crystal Mount (Biomeda, USA). Fluorescence images were captured using an EVOS fluorescence microscope (Thermo Fisher Scientific).

### Immunohistochemistry Staining

Protein expression in ocular tissues was analyzed by immunohistochemistry staining using paraffin-embedded sections. Primary antibodies against cluster of differentiation 3 (CD3) and cluster of differentiation 4 (CD4; Abcam), intercellular adhesion molecule-1 (ICAM-1), vascular cell adhesion molecule-1 (VCAM-1), Bcl-2-associated X protein (Bax), and B-cell lymphoma 2 (Bcl-2; Santa Cruz Biotechnology, USA) were used. Goat anti-mouse IgG conjugated to horseradish peroxidase (HRP) and goat anti-rabbit IgG-HRP (Santa Cruz Biotechnology) were used as secondary antibodies. Following deparaffinization and rehydration, the paraffin sections were processed according to Vectastain ABC HRP kit protocol (Vector Laboratories, USA). After blocking, sections were incubated overnight at 4°C with primary antibodies, followed by HRP-conjugated secondary antibodies. Finally, immunoreactivity was visualized with 3,3'-diaminobenzidine (Vector Laboratories), and nuclei were counterstained with hematoxylin [30].

### Periodic Acid–Schiff (PAS) Staining

PAS staining for detecting mucopolysaccharides and glycoproteins was performed using a commercial PAS staining kit (Sigma-Aldrich Chemical Co.), according to the manufacturer’s instructions [27].

### Western Blot Analysis

Western blot analysis was performed according to a previously described protocol [31] with slight modifications. Briefly, proteins were extracted using lysis buffer and quantified. Equal amounts of protein were separated *via* SDS-PAGE and transferred onto nitrocellulose membranes (GE Healthcare, USA). After blocking with 5% skim milk, the membranes were incubated overnight at 4°C with primary antibodies. The following day, the membranes were incubated at RT for 1 h with secondary antibodies, including goat anti-mouse and anti-rabbit IgG-horseradish peroxidase (Santa Cruz Biotechnology, Inc., USA), diluted at a 1:15,000 ratio. Protein bands were visualized using the SuperSignal West Pico PLUS chemiluminescent substrate (Thermo Fisher Scientific) and detected with the Fusion FX imaging system (Vilber Lourmat, France). The following primary antibodies were used: anti-TNF-β and anti-VEGF antibodies (Santa Cruz Biotechnology, Inc.), along with an anti-β-actin antibody (Bioworld Technology Inc.).

### Statistical Analysis

All results are expressed as mean ± standard deviation (SD). Statistical comparisons were made using one-way ANOVA followed by Tukey’s post hoc test in GraphPad Prism 8.0 (GraphPad Software Inc., USA). A *p*-value < 0.05 was considered statistically significant.

## Results

### Cytoprotective Effects of MMH against DED in Corneal Epithelial Cells *In Vitro*

**Fig. 1:**
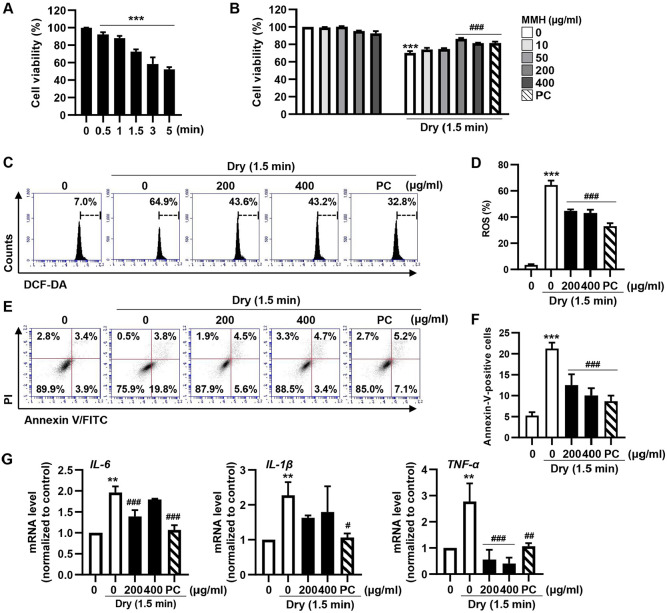
Cytoprotective effects of MMH in a dry eye-induced cellular model with hCECs. (**A** and **B**) Cell viability assessed *via* CCK-8 assay. (**A**) hCECs exposed to desiccation stress for 0, 0.5, 1, 1.5, 3, and 5 min. (**B**) hCECs treated with MMH at concentrations of 10, 50, 200, and 400 μg/ml, or pretreated with MMH for 1 h prior to 1.5 min of desiccation stress. (**C–G**) Cells pretreated with 200 or 400 μg/ml MMH for 1 h before air-drying. (**C** and **D**) Intracellular ROS levels detected *via* DCF-DA staining, followed by flow cytometry (**C**) and quantified (**D**). ROS levels measured 30 min after exposure to desiccation stress. (**E** and **F**) Apoptosis evaluated by Annexin V staining (**E**) and quantified (**F**). (**G**) Expression levels of proinflammatory cytokine mRNAs examined *via* qRT-PCR. Data expressed as mean ± SD (*n* = 3) **p < 0.01, ***p < 0.001 vs. control and ^###^*p* < 0.001 vs. Dry.

hCECs were used as an *in vitro* model to evaluate the protective effects of MMH against desiccation-induced DED. A 1.5-min air-drying period, which reduced cell viability to approximately 70%, was selected as the optimal condition for DED induction (Fig. 1A). Subsequently, the hCECs were treated with MMH at concentrations of 10, 50, 200, and 400 μg/ml (Fig. 1B). MMH treatment alone did not affect cell viability. However, pretreatment with 200 and 400 μg/ml MMH prior to DED induction significantly prevented the desiccation-induced reduction in cell viability (Fig. 1B). Furthermore, intracellular ROS levels were measured *via* DCF-DA staining. MMH pretreatment significantly ameliorated them (Fig. 1C and 1D). Additionally, Annexin V-FITC/PI staining was performed to examine the mode of cell death. The results demonstrated that MMH pretreatment alleviated desiccation-induced apoptosis in hCECs (Fig. 1E and 1F). This study further examined the expression of pro-inflammatory cytokines. As depicted in Fig. 1G, desiccation significantly upregulated both mRNA and protein levels of IL-6, IL-1β, and TNF-α, whereas MMH pretreatment effectively suppressed the expression of these inflammatory mediators in a dose-dependent manner. Collectively, these findings suggest that MMH protects hCECs from desiccation-induced damage, potentially by exerting antioxidant, anti-apoptotic, and anti-inflammatory effects.

### MMH Recovers Ocular Surface Damage and Tear Volume in a DED Rat Model

**Fig. 2:**
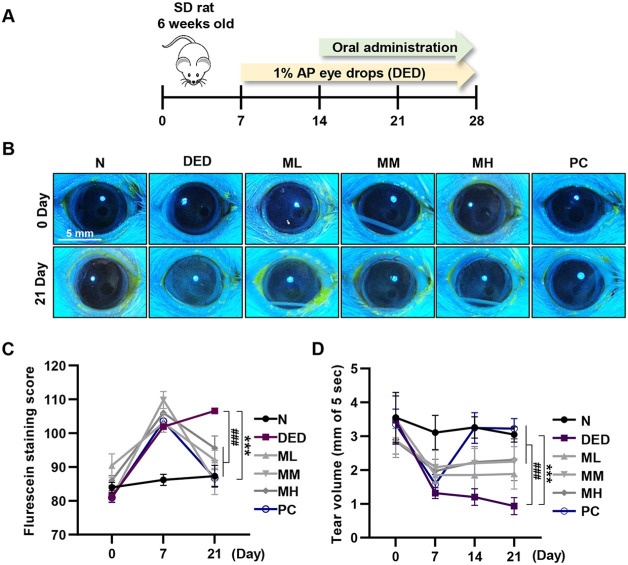
Establishment of a DED-induced animal model and evaluation of MMH efficacy. (**A**) Schematic illustration of *in vivo* experimental design. DED was induced by topical AP administration in SD rats for 7 days, followed by oral MMH administration for 14 days. MMH was administered at three doses: 50 mg/kg (ML), 100 mg/kg (MM), and 200 mg/kg (MH). (**B**) Corneal damage visualized *via* fluorescein staining. (**C**) Fluorescent area quantified using ImageJ software. (**D**) Tear volume was measured weekly using tear strips. Data expressed as mean ± SD (*n* = 3) ***p < 0.001 vs. normal and ^###^*p* < 0.001 vs. DED.

To evaluate the protective effects of MMH on the corneal epithelium and tear secretion. The *in vivo* model established to confirm the dry eye treatment effect of MMH is shown in Fig. 2A. Corneal damage was subsequently assessed by fluorescein staining. Fluorescein uptake was markedly increased in the DED group, indicating substantial corneal injury. However, MMH treatment significantly reduced the extent of fluorescein staining (Fig. 2B), and quantitative analysis demonstrated a greater reduction in the MM group (Fig. 2C). In addition, the tear secretion volume gradually decreased over time in the DED group (Fig. 2D). In contrast, MMH-treated groups exhibited a gradual increase in tear volume, with a more pronounced effect observed in the MH group, which was comparable to that of the positive control group. These results demonstrate the successful establishment of a DED model *via* AP treatment and suggest that MMH exerts a notable therapeutic effect in alleviating dry eye symptoms.

### Protective Effects of MMH on Corneal Integrity and Barrier Function in a DED Rat Model

**Fig. 3:**
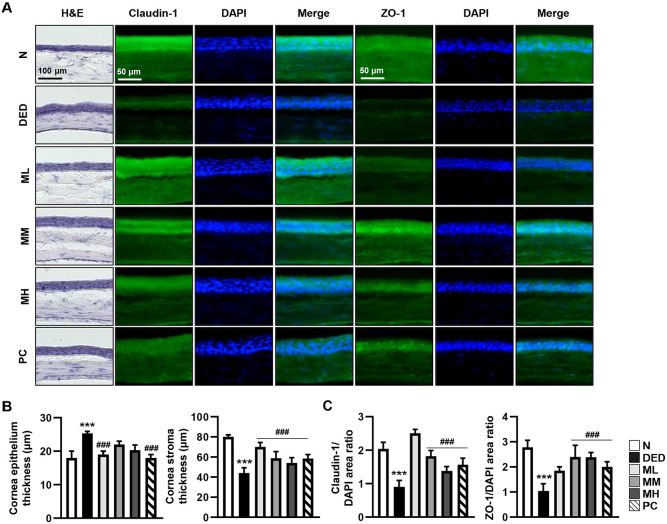
Protective MMH effects on corneal structures. Following 14 days of oral MMH administration, rat eyes were collected to assess corneal and retinal structural integrity. (**A**) H&E staining was performed on corneal sections to evaluate tissue morphology. Expression of tight junction proteins Claudin-1 and ZO-1 assessed *via* immunofluorescence staining using anti-Claudin-1 and anti-ZO-1 antibodies. Nuclei were counterstained with DAPI. (**B** and **C**) Morphological changes and fluorescein staining scores quantified using ImageJ software. Data expressed as mean ± SD (*n* = 3) ***p < 0.001 vs. normal and ^###^*p* < 0.001 vs. DED.

Histological changes in the cornea were assessed to evaluate the protective effects of MMH. H&E staining revealed that the corneal epithelial thickness was markedly increased, whereas endothelial layer was thinned in the DED group. The corneal surface also appeared irregular and disrupted. However, oral MMH administration effectively restored corneal structure and thickness comparable to the normal group, wherein the most pronounced effect was observed in the ML group (Fig. 3A). In addition, the expression of tight junction proteins was evaluated through immunofluorescence. In the DED group, claudin-1 and ZO-1 protein expression levels decreased significantly (Fig. 3B). Nevertheless, MMH treatment enhanced the expression of these proteins. Specifically, claudin-1 expression was significantly increased in the ML group, whereas ZO-1 expression was highest in the MM group (Fig. 3C).

### MMH Attenuates Ocular Surface Inflammation by Suppressing Inflammatory Cytokine Expression and Inflammasome Activation in a DED Rat Model

**Fig. 4:**
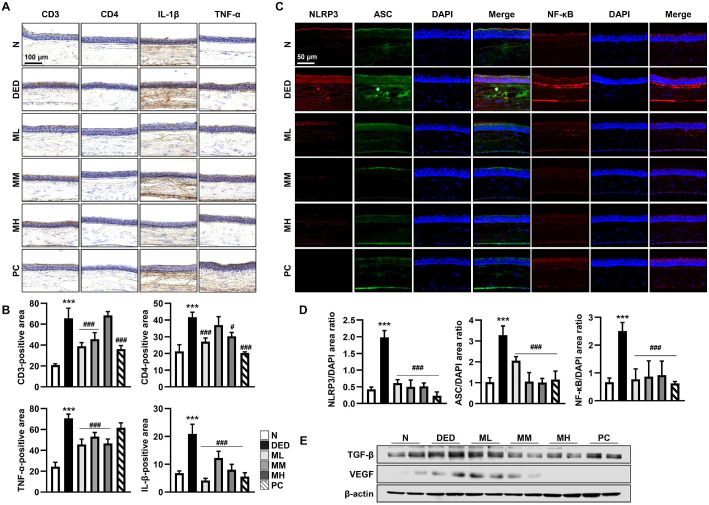
MMH protects against DED in the cornea through anti-inflammatory mechanisms. (**A**) Expression and localization of immune cell markers (CD3 and CD4) and inflammatory cytokines (IL-1β and TNF-α) in the cornea evaluated *via* immunohistochemical staining. (**C**) Expression of inflammasome complex proteins (NLRP3 and ASC) and the inflammatory mediator NF-κB examined *via* immunofluorescence using specific antibodies, with DAPI used for nuclear staining. (**B** and **D**) Staining intensity of each marker was quantified using ImageJ software. (**E**) Expression of additional inflammatory markers, TGF-β and VEGF, in the cornea measured *via* Western blotting. Data expressed as mean ± SD (*n* = 3) ***p < 0.001 vs. normal, #*p* < 0.05 and ^###^*p* < 0.001 vs. DED.

Subsequently, we investigated the mechanisms underlying the protective effects of MMH on the cornea. DED is known to disrupt the tear film and induce proinflammatory cytokine expression [32]. Therefore, immuno-histochemical staining was performed to evaluate cytokine expression in the cornea (Fig. 4A). In the DED group, CD3 and CD4 expressions, were significantly upregulated, along with the proinflammatory cytokines IL-1β and TNF-α. In contrast, MMH administration significantly reduced their expression, wherein the most pronounced effect was observed in the ML group (Fig. 4B). To further elucidate the underlying mechanisms, inflammasome-related proteins (NLRP3 and ASC) and NF-κB expression levels were examined using immunofluorescence staining (Fig. 4C). DED group markedly increased the expression of NLRP3, ASC, and NF-κB in corneal tissue, whereas MMH treatment significantly suppressed their expression, with the MM group demonstrating the strongest inhibitory effect (Fig. 4D). Furthermore, the expression of TGF-β and VEGF, key inflammatory mediators implicated in ocular disease progression, was notably decreased in the MM group (Fig. 4E). Collectively, these results indicate that MMH exerts anti-inflammatory effects against AP-induced corneal injury by modulating cytokine production and suppressing inflammasome activation in DED.

### MMH Alleviates Retinal Damage and Inflammation in a DED Rat Model

**Fig. 5:**
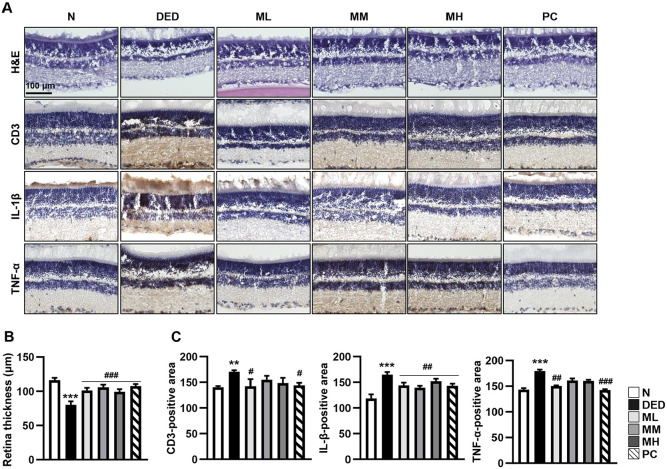
Anti-inflammatory and structural protective effects of MMH in the retina. Retinal tissues were harvested and fixed after 14 days of MMH administration. (**A**) Retinal morphology evaluated using H&E staining. Expression of the immune marker CD3 and inflammatory cytokines IL-1β and TNF-α detected *via* immunohistochemistry staining. (**B** and **C**) Retinal thickness and immunohistochemistry staining intensity quantified using ImageJ software. Data presented as mean ± SD (*n* = 3). ** *p* < 0.01, ***p < 0.001 vs. Normal; ^#^*p* < 0.05, ^##^*p* < 0.01 and ^###^*p* < 0.001 vs. DED.

Taking into consideration the secondary effects of tear film instability on posterior ocular tissues, including the retina, retinal changes were also investigated. H&E staining revealed that the DED group showed significantly reduced total retinal thickness and disrupted nuclear layer organization (Fig. 5A and 5B). MMH treatment alleviated these degenerative changes and preserved retinal morphology, with the most prominent protective effect observed in the MM group (Fig. 5A). Furthermore, the anti-inflammatory effects of MMH in the retina were evaluated. Immunohistochemical staining revealed that DED markedly increased CD3, IL-1β, and TNF-α expression in the retinal tissue. However, these proinflammatory markers were significantly reduced in MMH-treated groups, with the most prominent suppression observed in the ML group (Fig. 5A and 5C).

### MMH Protects the Lacrimal Glands from Inflammation and Apoptosis in a DED Rat Model

**Fig. 6:**
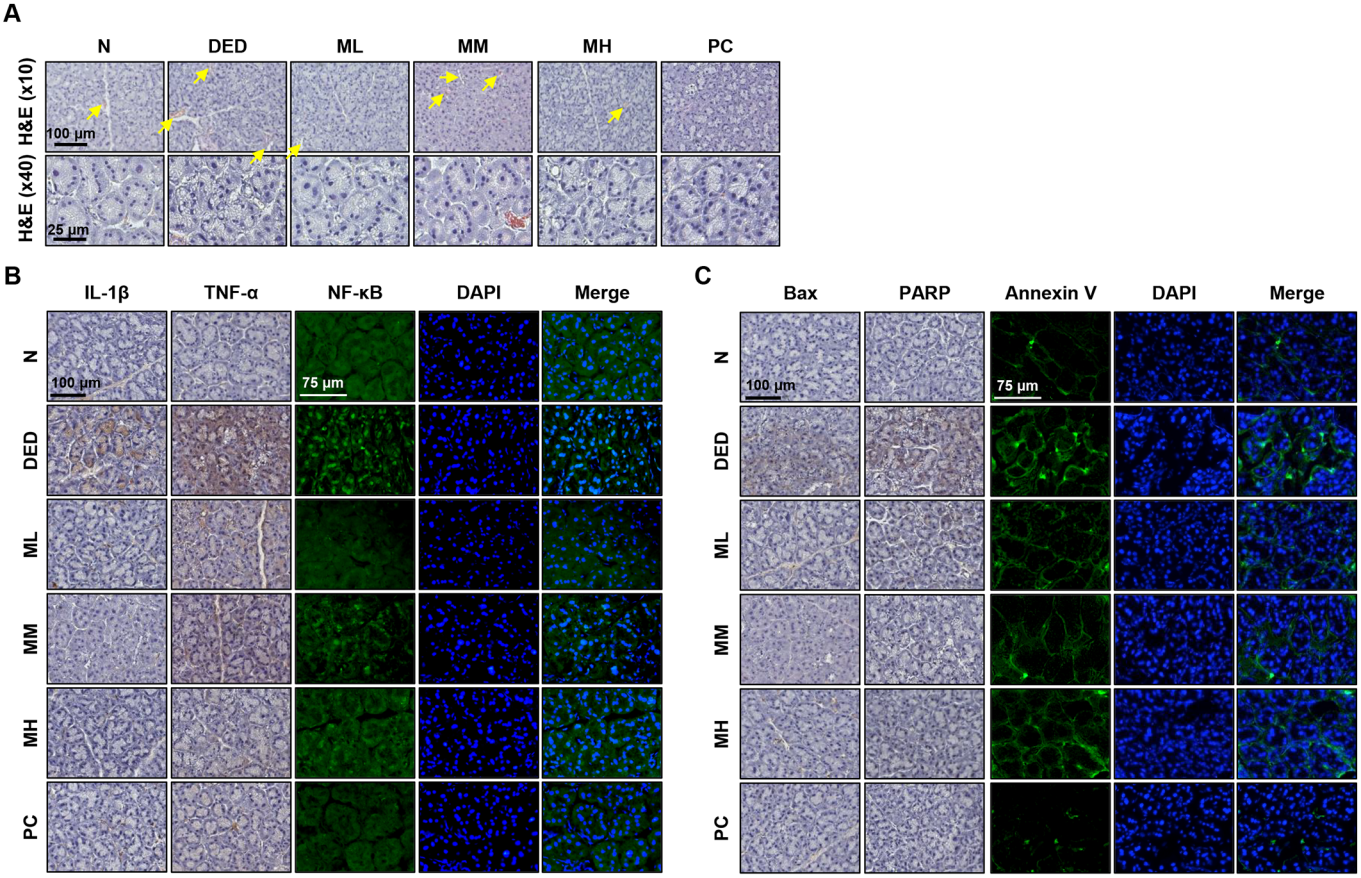
MMH exerts anti-inflammatory and anti-apoptotic effects in the lacrimal gland. Lacrimal glands were collected from rats following 14 days of oral MMH treatment. (**A**) Tissue morphology was evaluated *via* H&E staining. (**B**) Expression of inflammatory cytokines IL-1β and TNF-α analyzed through immunohistochemical staining; NF-κB expression assessed *via* immunofluorescence. (**C**) Apoptosis-related proteins Bax and PARP evaluated *via* immunohistochemical staining; apoptotic cells detected using Annexin V dye.

This study further assessed the protective effects of MMH on the lacrimal glands. Histological analysis using H&E staining revealed pronounced neovascularization (yellow arrow) in the lacrimal glands of the DED group (Fig. 6A). At higher magnification, extensive damage to the acinar structures was observed, including numerous pores and marked architectural disorganization. These pathological changes were alleviated by oral MMH administration, with the most pronounced protective effects observed in the ML group. To further evaluate the inflammatory response, IL-1β, TNF-α, and NF-κB expression levels were significantly elevated in the DED group. Conversely, IL-1β and TNF-α expression was most significantly suppressed in the MH group, whereas NF-κB expression was most suppressed in the ML group (Fig. 6B). Additionally, some of the apoptosis-related markers were also evaluated. Expression of the pro-apoptotic proteins Bax and PARP, as well as Annexin V immunostaining, significantly increased in the DED group and suppressed in all MMH-treated groups, with no substantial differences between doses (Fig. 6C).

### MMH Preserves Eyelid Glands and Goblet Cells in a DED Rat Model

**Fig. 7:**
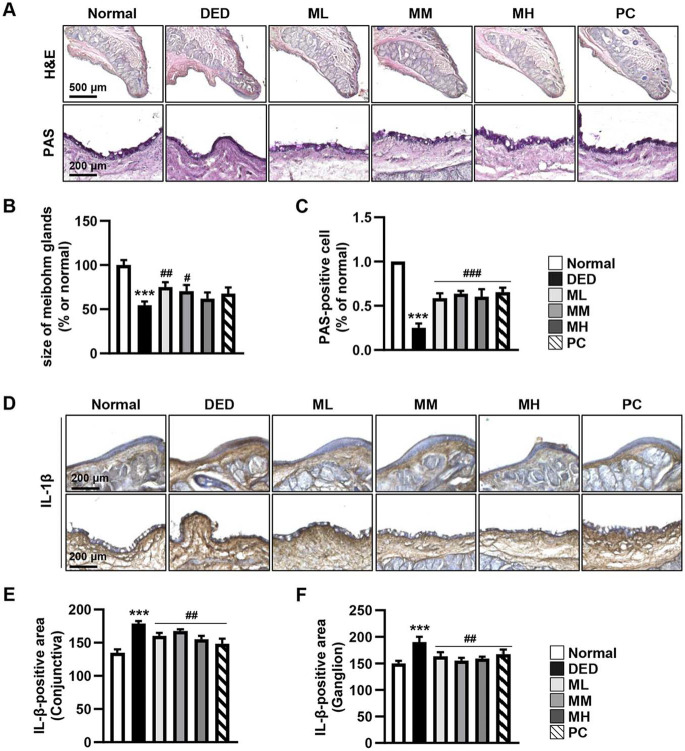
Protective effects of MMH on eyelid structure. Eyelids were harvested and fixed 14 days after MMH administration. (**A**) Structural abnormalities evaluated *via* H&E staining. Goblet cells within the conjunctival epithelium as visualized by PAS staining. (**B** and **C**) Size of meibomian glands and number of goblet cells quantified using ImageJ software. (**D**) Proinflammatory cytokine IL-1β expression assessed through immunohistochemical staining. IL-1β localization examined in the eyelid outer margin (top) and goblet cell layer (bottom). All parameters were quantified using ImageJ software and compared with the normal control group. Data presented as mean ± SD (*n* = 3). ***p < 0.001 vs. Normal; ^#^*p* < 0.05, ^##^*p* < 0.01 and ^###^*p* < 0.001 vs. DED.

Finally, the protective effects of MMH on eyelid tissue were histologically evaluated. H&E staining of the eyelids from DED group revealed a marked reduction in the size of the meibomian glands (Fig. 7A and 7B). Furthermore, PAS staining was performed to assess conjunctival goblet cells. The number of PAS-positive cells was significantly decreased in the DED group, whereas MMH treatment restored goblet cell density (Fig. 7A and 7C). No dose-dependent differences were observed. Immunohistochemical staining revealed a significant increase in IL-1β expression in the eyelids of the DED group (Fig. 7D–7F). However, oral MMH administration significantly suppressed IL-1β expression at all tested doses. Overall, these results demonstrate that AP-induced DED causes widespread structural and inflammatory damage across multiple ocular tissues, wherein oral MMH administration effectively protects the eyelids, conjunctiva, and other ocular structures.

## Discussion

DED is a complex and multifactorial ocular disorder whose global prevalence is rising, driven by environmental stressors such as urbanization and air pollution [10, 11]. Central to its pathogenesis are the phenomena of oxidative stress, inflammation, and apoptosis, which cause progressive damage to the ocular surface and adnexal tissues [18]. Therefore, therapeutic agents capable of simultaneously modulating these pathogenic processes are of great clinical interest.

To evaluate the therapeutic potential of MMH, this study utilized hCECs, which are directly exposed to the tear film and external environment, making them particularly susceptible to DED-related stress [33]. Consistent with previous studies [34, 35], our results indicated that desiccation stress significantly reduced cell viability, elevated ROS levels, and induced apoptosis in hCECs. In addition, desiccation stress markedly increased the secretion of proinflammatory cytokines, including IL-6, IL-1β, and TNF-α. Further supporting these findings, Wang *et al*. [33] demonstrated that gasdermin E, a key mediator of both apoptosis and pyroptosis, an inflammatory form of programmed cell death, contributes to DED pathogenesis by promoting inflammation *via* the pyroptosis pathway. Importantly, treatment with MMH effectively alleviated these deleterious effects. These observations validate the successful establishment of our *in vitro* DED model and highlight the potential of MMH as a protective agent for ocular surface cells. To further assess the *in vivo* efficacy of MMH, a DED animal model was established by administering AP eye drops to SD rats, in accordance with the Korean Ministry of Food and Drug Safety guidelines on functional foods for eye health. Successful DED induction was confirmed based on established criteria [36, 37], including a significant reduction in tear volume and increased corneal fluorescein staining.

The cornea is composed of several distinct layers: the epithelium, which serves as the outermost barrier that protects the eye from external insults, and the stroma, which forms the bulk of corneal thickness, contributing to its structural integrity through a collagen-rich matrix [38, 39]. For optimal visual function, the uniformity of corneal shape and thickness must be maintained to ensure proper light refraction. However, in our study, the corneal architecture in the AP-treated group demonstrated significant alterations compared to the normal control group. In particular, the expression of Claudin-1 and ZO-1, key tight junction proteins essential for maintaining corneal epithelial barrier function, was significantly reduced in the DED group. Previous studies have identified Claudin-1 and ZO-1 as critical markers of epithelial integrity [40,41], and their downregulation is widely used as an indicator of corneal epithelial damage [42]. Moreover, Yao *et al*. [43] reported that mice treated with anti-ZO-1 neutralizing antibodies exhibited aggravated alkali-induced corneal neovascularization, which was further associated with increased intracorneal infiltration of progenitor and inflammatory cells. Notably, in our study, the MMH-treated group exhibited a substantial restoration in Claudin-1 and ZO-1 expression compared to the DED group, suggesting that MMH confers a protective effect on corneal epithelial barrier integrity under desiccation-induced stress.

Based on the findings from our *in vitro* model, we further investigated the inflammatory responses associated with DED-induced corneal damage to elucidate the underlying mechanisms. Inflammation has been well-established as a central component in DED pathogenesis, as supported by numerous studies. This includes research by Stevenson *et al*. [44], who provided comprehensive evidence for the involvement of inflammatory processes. Similarly, Fu *et al*. [45] demonstrated that the total glucosides of paeony ameliorated ocular surface damage in Sjögren's syndrome mice by modulating gut microecology and maintaining immune homeostasis *via* the “gut-eye axis.” Similarly, Zhang *et al*. [34] employed proteomic analysis to explore the molecular mechanisms of corneal injury in DED and confirmed the role of inflammation. Their research further indicated that treatment with Qingxuan Runmu Yin decoction restored corneal health through its anti-inflammatory effects. Consistent with these findings, our results suggested that MMH exhibited a strong protective effect on the cornea by significantly suppressing the expression of immune cell markers (CD3 and CD4) and inflammation-associated adhesion molecules (ICAM-1 and VCAM-1). Notably, MMH also demonstrated a robust anti-inflammatory effect in the retina, as evidenced by retinal structure restoration and reduced inflammatory marker expression. Moreover, MMH treatment led to a marked decrease in the expression of NLRP3 and ASC, key components of the inflammasome complex, as well as NF-κB, a central mediator of inflammatory signaling. These findings highlight the anti-inflammatory properties of MMH and suggest that its effects may be mediated, at least partially, through NF-κB pathway inhibition. In addition, MMH suppressed the expression of pro-inflammatory cytokines TGF-β and VEGF, both of which are implicated in the development of various ocular diseases. Collectively, these results suggest that MMH exerts potent anti-inflammatory effects on the ocular surface and may serve as a promising therapeutic agent for DED treatment.

DED has been shown to cause extensive damage to ocular tissues, primarily as a result of elevated inflammatory mediators and increased tear film osmolarity. These pathological changes result in the generation of toxic tear components that can directly damage the conjunctiva, cornea, and lacrimal glands [46, 47]. Recent studies further report that DED promotes apoptosis in the lacrimal glands, thereby contributing to tissue dysfunction [48, 49]. In parallel, protein hydrolysates have been reported to inhibit apoptosis through the antioxidant activity of bioactive peptides [3, 4], which supports the therapeutic potential of such compounds. In accordance with these findings, the present study also demonstrated notable pathological changes in the lacrimal glands of the DED group. Specifically, neovascularization (indicated by yellow arrows) and severe damage to the acinar structures were observed. Furthermore, the expression levels of proinflammatory cytokines, including IL-1β, TNF-α, and the central inflammatory regulator NF-κB, were markedly upregulated. In contrast, the MMH-treated group exhibited substantial anti-inflammatory effects. Consistent with previous reports, DED induction significantly increased the expression of apoptotic proteins such as Bax and PARP, which indicated the activation of the apoptotic pathway. Concurrent with these findings, the expression of Annexin V, a marker that specifically stains apoptotic cells, was also elevated in the DED group. Importantly, MMH treatment effectively attenuated apoptosis in a concentration-independent manner, highlighting its potent anti-apoptotic potential.

Finally, we examined the eyelids, which function as an integrated unit in conjunction with the tear film to protect and lubricate the ocular surface [50]. The conjunctival epithelium of the eyelid contains goblet cells responsible for mucin secretion and houses the meibomian glands, which produce the lipid layer of the tear film [51, 52]. In this study, DED induction led to a marked reduction in the size of the meibomian glands, as well as a decrease in the area covered by PAS-stained goblet cells. In addition, IL-1β expression was significantly elevated in the eyelids of the DED group, indicating a pronounced inflammatory response. Conversely, MMH treatment substantially ameliorated these pathological changes, restored goblet cell and meibomian gland integrity, and reduced inflammatory marker expression. These findings suggest that MMH also exerts anti-inflammatory and protective effects in the eyelids, highlighting its therapeutic potential for DED treatment.

**Fig. 8:**
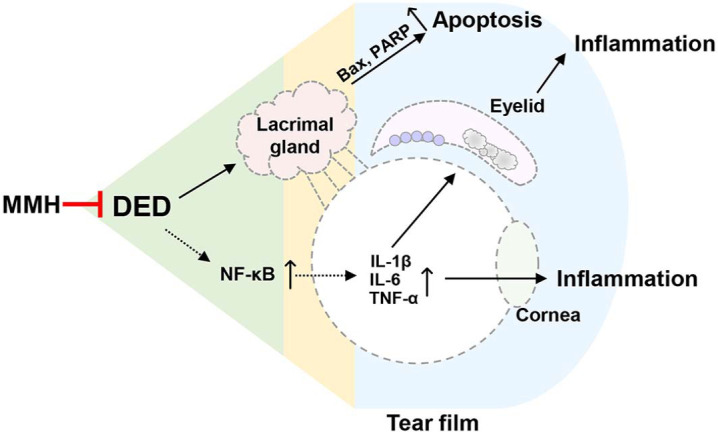
Schematic overview of the protective effects of MMH against DED. Oral MMH administration alleviates DED by reducing inflammation and apoptosis, restoring tear secretion, and preserving ocular tissue integrity (cornea, lacrimal gland, retina, and eyelid).

In conclusion, MMH demonstrated potent protective effects against dry eye disease by attenuating oxidative stress, apoptosis, and inflammation across multiple ocular tissues. MMH also demonstrated a broad spectrum of action by preserving epithelial barrier integrity, lacrimal gland function, goblet cells, and meibomian glands (Fig. 8). However, this study was conducted over a relatively short period, and further investigations are warranted to elucidate the precise molecular mechanisms of MMH, including the identification of its active constituents. Despite these limitations, our findings strongly support the therapeutic potential of MMH as a novel and promising candidate for the treatment of dry eye disease.

## Figures and Tables

**Table 1:** Primer sequences used for qRT-PCR analysis.

Gene	Primer	Primer sequences (5’-3’)	Annealing temperature (°C)
IL-6	F	AGA CAG CCA CTC ACC TCT TCA G	60.0
	R	TTC TGC CAG TGC CTC TTT GCT G	60.0
IL-1β	F	GGG CCT CAA GGA AAA GAA TC	55.4
	R	TTC TGC TTG AGA GGT GCT GA	55.4
TNF-α	F	CAG AGG GCC TGT ACC TCA TC	59.5
	R	GGA AGA CCC CTC CCA GAT AG	59.5
GAPDH	F	CCA TGT TCG TCA TGG GTG TGA	58.4
	R	CAT GAG TCC TTC CAC GAT ACC A	58.2
